# IL-27 regulation of innate immunity and control of microbial growth

**DOI:** 10.2144/fsoa-2020-0032

**Published:** 2020-06-17

**Authors:** Jessica M Povroznik, Cory M Robinson

**Affiliations:** 1 Department of Microbiology, Immunology & Cell Biology, West Virginia University School of Medicine, Morgantown, WV 26506, USA; 2 Vaccine Development Center, West Virginia University Health Sciences Center, Morgantown, WV 26506, USA

**Keywords:** antigen presenting cells, autophagy, bacterial, innate immunity, interleukin-27, lysosomal acidification, myeloid-derived suppressor cells, neonates, oxidative burst

## Abstract

IL-27 is a pleiotropic cytokine capable of influencing both innate and adaptive immune responses. With anti- and pro-inflammatory activity, IL-27 exerts its opposing effects in a cell-dependent and infectious context-specific manner. Upon pathogenic stimuli, IL-27 regulates innate immune cells, such as monocytes, dendritic cells, macrophages and neutrophils. Immune responses involving these innate cells that are negatively regulated by IL-27 signaling include inflammatory cytokine production, phagolysosomal acidification following phagocytosis, oxidative burst and autophagy. IL-27 signaling is crucial in maintaining the subtle balance between Th1 and Th2 immunity, in which protective inflammation is upregulated within the early stages of infection and subsequently downregulated once microbial growth is controlled. The immunomodulatory effects of IL-27 provide promising therapeutic targets for multiple disease types.

## Lay abstract

A primary role of IL-27 is to communicate between various immune cells to initiate different immune responses. Among these responses are those involved with destroying and eliminating microbial pathogens and then turning off inflammatory responses when the infectious threat has been resolved. IL-27 possesses both anti- and pro-inflammatory activity that varies with context, immune cell and pathogen stimulus. Depending on the precise formula of these details, there are important implications for IL-27 in disease outcomes. As such, harnessing or opposing IL-27 activity may have the potential to treat a variety of infectious diseases.

IL-27 influences immunity in divergent ways that affect both the innate and adaptive arms of the immune system. IL-27 is a heterodimeric cytokine comprised of *EBI3* and IL-27p28 (commonly abbreviated to p28) proteins [1,2]. IL-27 is categorized as a type-1 cytokine defined by shared structural motifs, such as the four-helix bundle and the hematopoietin-receptor domain, as well as similar signaling properties among both the ligands and receptors that are common to other members of the IL-12 family, including IL-6 and IL-23 [3]. The receptor complex for IL-27 consists of WSX-1 (also referred to as IL-27Rα, or T cell cytokine receptor) and the glycoprotein, gp130 [4]. Upon interaction with the receptor, IL-27 signals primarily through JAK-STAT and p38 MAPK/ERK pathways [5,6]. However, the NF-κβ pathway has also been shown to initially act in concert with STAT signaling through the stimulation of essential innate immune response facilitators: these include Toll-like receptors (TLRs) or tumor necrosis family (TNF) receptors, type I and II interferons (IFNs) and the complement cascade, to either sustain (through TLR signaling) or decrease (via complement signaling) the production of IL-27p28 produced by antigen-presenting cells (APCs) [7–10]. The adaptor protein, MyD88, is a key component in TLR signaling that is also known to play a role in inducing IL-27 transcription and its involvement in these pathways can be described in further detail in these excellent reviews [11,12].

Initiation of these signaling cascades leads to diverse biological outcomes involved in cell survival and proliferation, as well as transformation of myeloid-derived cells, tissue inflammation, cell death and suppression of inflammatory responses. IL-27 was first described as a pro-inflammatory cytokine paramount to cell-mediated Th1 differentiation and increased IFN-γ production through IL-12 stimulation and activation of the transcription factor T-bet [1,6,13,14]. However, since that time, numerous studies have shed light on the intrinsically nuanced facets of IL-27 signaling whereby the cytokine has revealed immunosuppressive capabilities [15–19]. This includes direct inhibition of pro-inflammatory cytokines and various T cell subsets (i.e., CD4^+^ T cells, Th17 cells and Foxp3^+^ Treg cells) or indirect antagonism through increased production of the canonical anti-inflammatory cytokine, IL-10 [13,17,20–24]. After decades of research, we are now understanding that the differences in the biological outcomes surrounding IL-27 signaling may be stimulus-specific, immune-cell type-dependent and influenced by the concentration in the local micro-environment. Owing to its ability to both initiate and suppress inflammation, IL-27 is a critical regulator in maintaining the subtle balance between Th1 and Th2 responses.

While IL-27 signaling has proven influences in both the innate and adaptive immune responses and acts as a bridge between the two systems, this review will focus primarily on the effects of IL-27 on innate immune cells and how these interactions impact control of bacterial growth. This will include discussion of the underlying mechanisms involved with control of multiple bacterial species and how these processes vary among distinct innate immune cells and nonimmune cells during different models of *in vivo* and *in vitro* infection of human cells and rodent populations. Furthermore, this review will address some of the clinical implications of IL-27 signaling among different pathological states in young and aged populations, including sepsis, tuberculosis, neurological diseases and HIV. Recent and ongoing research points to the development of IL-27 modulation as a viable therapeutic target.

## IL-27 regulation in innate immune cells

### Antigen-presenting cells

Professional APCs, such as monocytes, macrophages and dendritic cells (DCs), are all common sources of IL-27 expression. However, these cells not only synthesize IL-27, but are also equipped to respond to IL-27 in an autocrine and paracrine manner. This response includes production of a variety of anti- or pro-inflammatory cytokines and chemokines in a pathogenic stimulus- and cell-type-dependent manner. For example, upon exposure to IL-27, both macrophages and DCs decrease production of TNF-α [25–27], while monocytes increase TNF-α expression following exposure to both IL-27 and lipopolysaccharide (LPS) [28]. Furthermore, Guzzo and colleagues demonstrated that the latter required NF-κβ- and STAT3-dependent upregulation of TLR4 [29]. Jung and colleagues showed that the timing of IL-27 signaling can also be critical in shaping the response. DCs differentiated from human monocytes in the presence of IL-27 exhibited improved antigen processing, enhanced IL-12 production and increased stimulation of T cell differentiation [30].

In conjunction with a direct decrease of pro-inflammatory cytokine production, IL-27 further exerts anti-inflammatory effects in macrophages by promoting increased expression of the anti-inflammatory cytokine, IL-10 [31]. Furthermore, in a murine model of oral tolerance, IL-10 production was preceded by an IL-27 increase in DCs from ovalbumin-fed mice, suggesting a direct influence of IL-27 [32]. In line with the immunosuppressive effects of IL-27, additional evidence also suggests that IL-27 negatively regulates APC function in DCs with consequences to promotion of a Th1 response and IFN-γ production, an effect that is observed concomitant with a reduced DC pro-inflammatory response [25]. However, following LPS stimulation in monocytes, IL-27 generates the opposite effect, with decreased IL-10 production [28]. The association between IL-27 expression and IL-10 secretion was first demonstrated with a *WSX-1* knockout mouse model of toxoplasmic encephalitis [20]. The interaction between IL-27 and IL-10 was also demonstrated in experimental autoimmune encephalomyelitis, a model commonly engineered in rodents and other small animals to study multiple sclerosis [33]. In these initial studies, pathological assessment found a correlation between both diminished levels of IL-27 and IL-10 in which mice deficient of the IL-27 receptor exhibited excessive inflammation; this effect was attenuated when mice were given exogenous IL-27 that increased production of IL-10 from effector T cells [20,33]. These and additional studies further validated the role of IL-27 as a promoter of IL-10 production from Th1, Th2, Th17 and Treg cells [34–36].

It is also important to note that professional APCs are not the only cellular sources of IL-27; neutrophils, microglial cells, myeloid-derived suppressor cells (MDSCs) and plasma cells, also either respond to and produce the cytokine or co-express p28 and EBI3 protein [37–42]. Nonimmune cells that influence the innate immune response such as endothelial and epithelial cells, as well as fibroblasts, have been shown to express IL-27 genes [7,43,44]. However, whether or not these cells release active protein remains to be demonstrated.

### Microglial cells

Acting as the primary immune cell of the CNS, microglia possess similar phagocytic properties as those of macrophages in the periphery. Microglia, similarly to other innate immune cells, can both secrete and respond to IL-27. In human brains with lesions caused by multiple sclerosis, it has been shown that the pro-inflammatory cytokine environment increases the production of IL-27 levels from microglia [45]. In contrast, in LPS-stimulated murine microglia, IL-27 enhanced the production of pro-inflammatory cytokines as well as neuroprotective neurotrophic factors like NGF, BDNF and GDNF [40]. However, other *in vitro* studies with murine cells demonstrated immune suppressive effects. Specifically, IL-27 suppressed oncostatin M (an IL-6 cytokine family member) induction of TNF-α and iNOS expression in microglial cells through inhibition of the NF-κβ pathway [46].

### Neutrophils

Neutrophils are a critical first line of defense in the innate immune response. Neutrophils exposed to IL-27 acutely increased their production of the pro-inflammatory cytokines IL-1β and TNF-α [47]. However, in the absence of IL-27 signaling in adult mouse models of sepsis (*WSX-1* knockout), increased neutrophil levels and corresponding reactive oxygen species (ROS) were demonstrated, consistent with reduced bacterial burdens. These findings suggest that IL-27 negatively regulates neutrophil recruitment, thereby increasing bacterial loads [37]. Similar findings have been documented in human neutrophils, wherein exposure to IL-27 diminished LPS-induced ROS production, as well as decreased neutrophil adhesion [48]. The primary caveat to this explanation is that IL-17, a cytokine known to promote neutrophil chemotaxis, is antagonized by IL-27 [22,49]. Thus, the influence of IL-27 on the increased neutrophil response in *WSX-1* knockout models may operate indirectly through an influence on IL-17 levels.

## IL-27 regulation in non-APC cells affecting innate immunity

The IL-27 receptor is also expressed on non-APC cells, including epithelial and endothelial cells, as well as fibroblasts. These cells play an active role in innate immunity, as they are known to stimulate immune cells involved in early-stage inflammation [50–52]. Specifically, it has been reported that IL-27 receptor expression is upregulated in intestinal epithelial cells during inflammation and bacterial infection, whereby IL-27 promoted enhanced barrier function through increased epithelial cell proliferation and restoration [53]. Since epithelial (and endothelial) barrier disruption acts as an initiator of a pro-inflammatory response during infection, Diegelmann and colleagues show that IL-27 has a role in limiting intestinal inflammation. Moreover, they demonstrated that IL-27 induced a protective antibacterial effect that was mediated through p38 and STAT3 signaling. As well, the beneficial antibacterial and anti-inflammatory effects of the IL-27-induced gene, *IDO1*, were mediated through STAT1 signaling [53].

In primary human umbilical vein endothelial cells, stimulation by IL-27 resulted in increased MHC II expression requiring *de novo* gene expression [54]. This further bolstered their ability to act as noncanonical APCs. Furthermore, IL-27-treated human umbilical vein endothelial cells also secreted CXCL9, CXCL10 and CX3CL1 [54]. These chemokines are classically mediated by IFN-γ and are largely involved with leukocyte adhesion and diapedesis during the innate inflammatory response [55]. Interestingly, IL-27 is also capable of inducing the chemokine CXCL10 in fibroblasts [56]. More specifically, Dong *et al.* demonstrated an association between increased IL-27 and CXCL10 levels in human lung fibroblasts of patients with the lung-related diseases, chronic obstructive pulmonary disease and pulmonary tuberculosis. The mechanism underlying the clinical manifestations was determined to be a synergistic effect between IL-27 and TNF-α, through which IL-27 expression enhanced TNF-α-induced phosphorylation of p38 MAPK and Akt.

## Microbial regulation of IL-27 & consequences during infection

Gram-positive and gram-negative bacteria as well as microbial stimuli such as TLR agonists LPS, CPG, poly(I:C) and others, induce expression of p28 and EBI3 in myeloid cells [5]. However, pathogen-specific influences on regulation of IL-27 have been delineated and this has implications in the balance between Th1 and Th2 immunity. For example, a variety of nonpathogenic gram-negative bacteria primed human DCs for increased IL-27 expression compared with commensal gram-positive bacteria [57]. This was consistent with the ability to initiate a polarized Th1 response that was also dependent on IL-12 and in contrast to gram-positive bacteria [57]. It has been suggested that IL-27 gene expression is regulated principally by TLR4 and not TLR2; however, that work did not incorporate gram-positive bacteria or known TLR ligands [58]. IFN-γ enhances and sustains LPS-mediated IL-27p28 transcriptional activation through IRF1 and 8 binding [59]. There are also roles for other intracellular pattern recognition receptors such as TLR3, 7 and 9 that regulate IL-27 gene expression in MyD88-dependent and independent manners through the induction of type I interferon signaling and IRF3 activation [60].

When IL-27 signaling is blocked during bacterial infection, a more pro-inflammatory response can be initiated through downregulation of IL-10 and subsequent up-regulation of Th1 cytokines to stop pathogen replication [61]. As described more below, IL-27 also directly influences pathways involved with microbial clearance. However, some studies have reported reduced inflammation upon IL-27 blockade [62–64]. Since bacteria and bacterial-derived components drive inflammation, an explanation for these findings is that lower IL-27 levels improve control of bacteria and thus reduced inflammation follows. In consummation, the mechanisms associated with IL-27 blockade upon bacterial infection are associated with increased bacterial killing and therefore decreased bacterial burden within the host cells. This ultimately improves survival and related outcomes for the host [41,61,62]. Although infection by *M. tuberculosis* is a noted exception. Because this is a chronic infection, the inflammatory response associated with prolonged absence of IL-27 signaling, while successful at improving control of bacterial growth, resulted in pathological and lethal tissue damage [26].

In contrast to most bacterial-induced infections, parasitic infections caused by helminths and protozoa, which typically trigger Th2 immunity, have demonstrated different outcomes as a result of IL-27 blockade. In a seminal study by Villarino and colleagues, IL-27-deficient mice infected with the parasite *Toxoplasma gondii* were able to control the growth of the parasite, but ultimately succumbed to the tissue pathology that resulted from excessive T-cell mediated inflammation [19]. Similar studies have reported lethal, exacerbated inflammation in IL-27-deficient mice in response to additional parasitic infections [16,65,66].

IL-27 also elicits pleiotropic effects during viral infection. Unlike bacterial-induced outcomes, but more similar to those of parasites, the harmful effects of viral infections are amplified in the absence of IL-27 signaling. For example, a 2014 study by de Almeida Nagata showed that viral-infected *WSX-1*-deficient mice administered neutralizing antibody for IL-17 (hypothesized to bolster IL-27 signaling, as IL-17 is a known antagonist of IL-27) showed reduced infiltration of DCs and neutrophils to infected tissue. This reduced cellular infiltration was associated with decreased tissue pathology and increased viral clearance [36]. In line with this, Liu *et al.* showed that treatment with IL-27 at peak virus load in a murine model of influenza, resulted in amelioration of lung pathology, reduced leukocyte infiltration and improved survival that was independent of viral clearance [67]. In contrast, when IL-27 was administered early after virus infection, viral clearance was impaired and increased immunopathology ensued [67]. This highlights the importance of timing in IL-27 signaling on the interplay of microbial clearance and maintaining the balance between protective and detrimental innate immunity.

## IL-27 & control of bacterial growth in innate immunity

**Figure 1.**
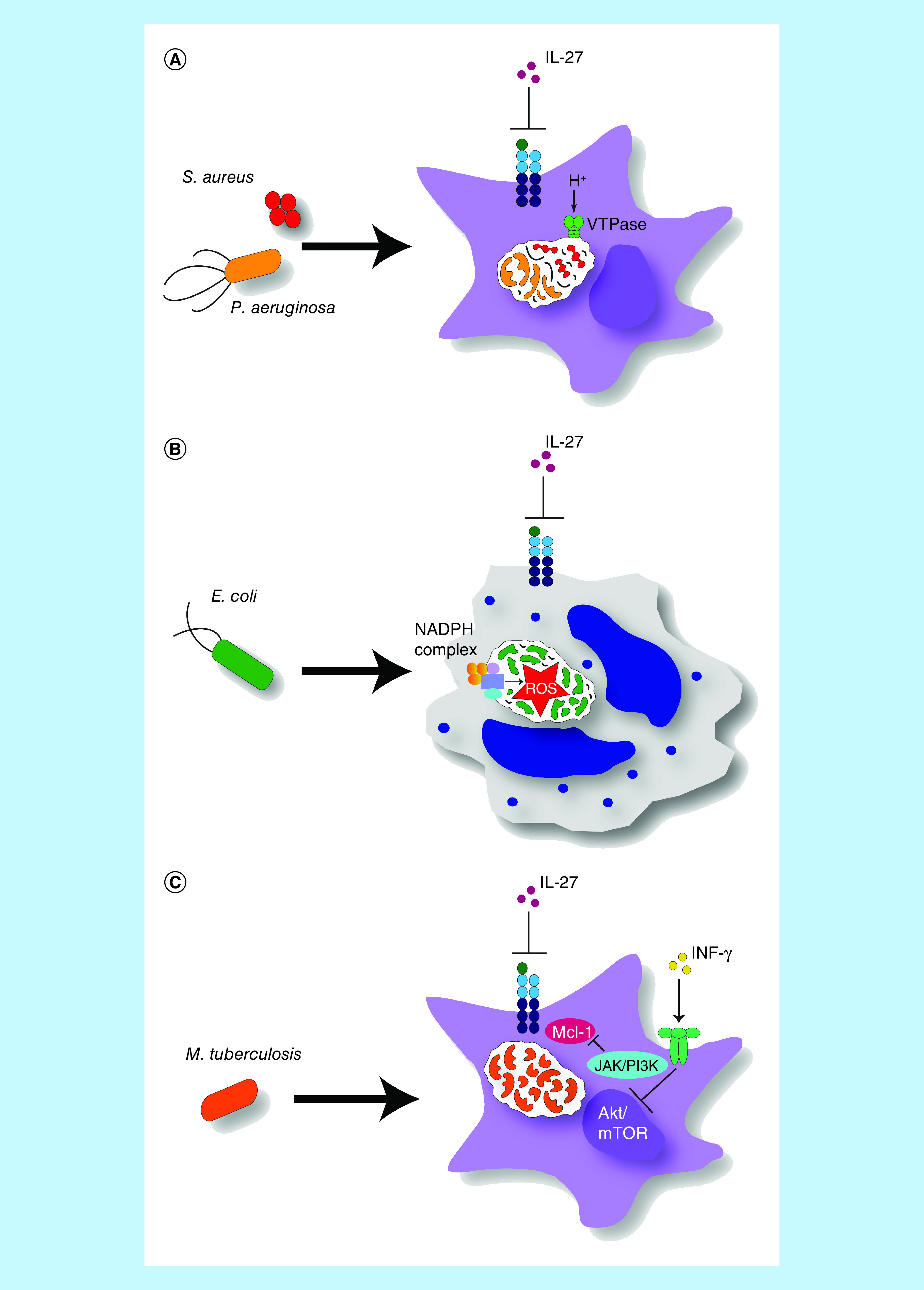
IL-27 signaling negatively regulates innate immune responses that control bacterial growth. **(A)** Lysosomal acidification. Upon inhibition of IL-27 signaling, macrophages injected with either *Pseudomonas aeruginosa* or *Staphylococcus aureus* upregulate expression of V-ATPase, the proton pump which enables acidification of the lysosome. **(B)** Oxidative burst. When IL-27 signaling is abrogated in neutrophils exposed to *Escherichia coli* or LPS, there is increased production of ROS within the cell, indicative of oxidative burst activity. ROS are generated via the NADPH complex and create an antimicrobial environment. **(C)** Autophagy. Blocking IL-27 signaling in macrophages exposed to *Mycobacterium tuberculosis* promotes IFN-γ-induced autophagy, which occurs through simultaneous inhibition of Akt/mTOR signaling via JAK/PI3K and increased inhibition of Mcl-1 also through JAK/PI3K. ROS: Reactive oxygen species; V-ATPase: Vacuolar H^+^-ATPase.

IL-27 signaling impairs control of bacterial growth through multiple mechanisms associated with the innate immune response. For example, Jung and colleagues demonstrated that IL-27 can impede bacterial growth (e.g., *Pseudomonas aeruginosa* and *Staphylococcus aureus*) by inhibiting the phagolysosomal pathway (Figure 1A) [68]. Key findings from this study showed that IL-27 decreased expression of molecules that are recruited to late endosomes and lysosomes. Most notably, this includes vacuolar H^+^-ATPase (V-ATPase) in macrophages. V-ATPases are multisubunit protein complexes that utilize ATP hydrolysis to promote acidification of intracellular phagosomal/lysosomal compartments [69]. Reduced levels of V-ATPase resulted in diminished lysosomal acidification that is required to eradicate bacterial pathogens internalized through phagocytosis [69,70]. The failure of the lysosome to properly acidify in turn led to reduced cathepsin activation further compromising the antimicrobial environment [71]. Moreover, lysosomal acidification and subsequent antigen degradation is necessary in order to sustain macrophage-induced phagocytosis [72]. Thus, V-ATPase blockade by IL-27 compromises destruction of bacteria by the innate immune response.

Additionally, IL-27 signaling can influence control of bacterial growth through the inhibition of autophagy in macrophages. This requires activation of the JAK, PI3K, Akt and mTOR signaling pathways (Figure 1B) [9]. A study by Sharma *et al.* reported that endogenous IL-27 blocked IFN-γ-induced autophagy which resulted in limited phagosomal maturation and therefore enhanced survival of *Mycobacterium tuberculosis* in infected macrophages in an mTOR-dependent manner. It is of note that mTOR is a negative regulator of autophagy [73]. Additionally, IL-27 has been shown to increase expression of Mcl-1, a member of the Bcl-2 protein family that functions to inhibit autophagy [74], through the PI3K pathway.

Furthermore, IL-27 has been shown to affect the process of oxidative (or respiratory) burst in granulocytes (Figure 1C) [37]. Oxidative burst is a crucial protective mechanism used by different immune cells, in which NADPH generates ROS upon activation of certain types of surface receptors that assist in host cell antimicrobial defense [75]. Wirtz *et al.* showed IL-27 to be a negative regulator of oxidative burst in neutrophils in their murine model of septic peritonitis. *EBI3*-deficient mice displayed a decreased bacterial load consistent with an increased oxidative radical production [37].

## IL-27 regulation in innate immunity of the neonatal population

Relative to adults, IL-27 levels are elevated in the human neonatal cells and mice. This was first reported by Kraft and colleagues [76]. They demonstrated that cord blood-derived macrophages expressed significantly higher levels of IL-27 genes and secreted more cytokine relative to adult macrophages [76,77]. In a murine model, they also reported similar findings of increased levels of IL-27-producing macrophages, increased splenic IL-27 gene expression and more abundant serum cytokine [41,76]. IL-27 is likely beneficial during pregnancy as fetal trophoblasts need to implant themselves into the uterus without activating the maternal immune response. Therefore, increased IL-27 production during fetal development is thought to confer an immunosuppressive effect that promotes immunological tolerance at the maternal–fetal interface and thus a successful pregnancy. Indeed, IL-27 has been shown to be expressed by trophoblasts invading the decidual tissue at all stages of pregnancy [78]. Expression of IL-27 was lower in deciduas of patients with unexplained recurrent miscarriage compared with control subjects [79]. Furthermore, IL-27 inhibited IL-17 production and promoted IL-10 production from CD4^+^ T cells isolated from decidual tissue taken from patients with recurrent miscarriage [79]. Collectively, this suggests that IL-27 makes important immunoregulatory contributions at the fetal–maternal interface that support successful pregnancy.

Progesterone has been shown to induce expression of IL-27 genes and the concentration of this hormone is highest in the third-trimester of pregnancy [76,80]. Although speculative, a possible advantage of elevated levels of IL-27 *ex utero* can be identified. As a neonate becomes colonized with commensal organisms in the first days of life, IL-27 may promote tolerance and avoidance of conditions such as necrotizing enterocolitis. However, increased levels of IL-27 shortly after birth are also associated with detrimental effects during infection [76]. Kraft *et al.* also showed that normally elevated levels of IL-27 in neonates led to reduced production of IFN-γ by CD4^+^ T cells stimulated *in vitro*, as well as diminished control of bacterial growth; neutralization of IL-27 reversed these effects, promoted enhanced IFN-γ production by CD4^+^ T cells and improved the ability of neonatal macrophages to control bacterial growth [76]. Further studies by Jung and colleagues demonstrated that increased levels of IL-27 in neonatal macrophages dose-dependently regulated IDO expression, a known suppressor of T-cell responses, through STAT1 and STAT3 signaling [77].

Similarly to macrophages, MDSCs have recently been found to be abundant producers of IL-27 in the spleen and blood of murine neonates [41,64]. MDSCs have potent immunosuppressive function and can be categorized into two subsets: monocytic or granulocytic. Human CD66^+^CD33^+^C14^-/Lo^MHC-II^-^ granulocytic MDSCs also express IL-27 genes (Unpublished data). While initial research established MDSCs as key regulators of tumor micro-environments and cancer progression [81], recent studies have begun to elucidate the role of MDSC-derived IL-27 in neonatal immunity during infection [41,64]. MDSCs compromised macrophage control of bacterial growth during co-culture and this was partially dependent on IL-27 [41]. Therefore, IL-27 may represent a new effector mechanism of immune suppression by MDSCs and these cells may be important contributors to the increased susceptibility to infection of the neonatal population.

## Clinical implications of IL-27 & its role in infectious disease

### Sepsis

In a mouse model of polymicrobial sepsis that was induced by cecal ligation and puncture, multiple IL-27-mediated consequences have been documented [62]. Specifically, Bosmann *et al.* demonstrated through both genetic manipulation of IL-27 signaling (e.g., *WSX-1* knockout), as well as cellular neutralization of the IL-27 receptor, reduced bacterial burden, reduced pro-inflammatory cytokine release, increased oxidative bursts in macrophages and increased survival outcomes in mice [62]. These findings are consistent with increased levels of IL-27 mRNA and protein found in humans with bacterial-induced sepsis. In these same patients, increased IL-27 levels are also associated with poor clinical outcomes [47]. In a study by Rinchai and colleagues, whole blood samples analyzed from *Burkholderia pseudomallei*-induced septic patients showed that increased IL-27 levels were primarily sourced from monocytes, macrophages and neutrophils [47]. Similar findings were observed with blood from healthy donors stimulated *in vitro* with *B. pseudomallei*. *In vitro* experimentation of the *B. pseudomallei*-infected human cells further demonstrated that administration of exogenous IL-27 increased bacterial survival through a reduction of the oxidative burst and increased levels of the pro-inflammatory cytokines, TNF-α and IL-1β. In contrast, IL-27 reduction *in vitro* through neutralization of IL-27 with a soluble receptor prior to bacterial infection, displayed increased oxidative burst, decreased IL-1β levels and therefore decreased bacterial survival [47].

### Tuberculosis

Although *M. tuberculosis* (Mtb) has been demonstrated to engage with both innate and adaptive immune cells [82], researchers have long questioned the mechanisms by which Mtb evades immune clearance. Pearl and colleagues found that IL-27 signaling opposes control of Mtb growth through a downregulation of IFN-γ and T-bet in CD4^+^ Th1 T cells [83]. Interestingly, the researchers noted in *WSX-1*-deficient mice, that although the frequency of IFN-γ-producing T cells had not decreased, the amount of IFN-γ produced per cell had declined. They suggested that the correlation between the decrease in IFN-γ-producing T cells and the increase of lymphocytes to the infected area (e.g., granuloma) in *WSX-1*-deficient mice attributed to the improved control of Mtb [83]. This is in contrast to other Mtb disease models in which IL-27 inhibition resulted in enhanced inflammation and subsequent immunopathological damage [26]. Hölscher *et al.* showed enhanced IFN-γ and TNF-induced inflammation as well as an enhanced ability of macrophages to control Mtb growth. However, *WSX-1*-deficient mice succumbed to the excessive pro-inflammatory response. Robinson and Nau showed IL-27 modulation of human macrophages with improved control of Mtb growth following neutralization of IL-27 with a soluble receptor (sIL-27R). This response required the addition of IL-12 and was consistent with an enhanced pro-inflammatory response [84].

### Nervous system infections

With many systemic infectious diseases, there is a possibility of the pathogen, bacterial, parasitic, viral, or other, to cross from the peripheral circulation through the blood–brain barrier (BBB) and into the brain parenchyma, which encapsulates the control center of the central nervous system (CNS). The BBB is a physical barrier consisting of specialized endothelial cells, tight junctions, pericytes and the end-feet of astrocytes [85]. Microbial pathogens have evolved a multitude of mechanisms through which they can cross the BBB and lead to numerous neurological sequelae that are often secondary to an initial infection within the periphery [86,87]. Once inside the CNS, immunopathological damage from the pathogen is often irreversible, leading to neuronal cell death and subsequent motor and/or cognitive impairment. In a study by de Aquino and colleagues, the authors infected mice with the JHM strain of murine hepatitis virus as a model of acute encephalomyelitis and observed an increase of IL-27p28 mRNA transcripts and IL-10 levels, as well as reduced viral clearance and increased demyelination of the brain tissue. All of these effects were attenuated in a JHM strain of murine hepatitis virus-infected mice deficient for *WSX-1* and were also accompanied by increased IFN-γ production from CD4^+^ and CD8^+^ T cells, suggesting a detrimental immunosuppressive role for IL-27 [88].

IL-27 can also influence infections that impact the peripheral nervous system (PNS) [89]. Roewe *et al.* utilized an LPS-induced murine model of endotoxic shock to recapitulate a similar pathology comparable to human sepsis. In their study, TLR4 receptors found on macrophages were activated with LPS resulting in an increased production of IL-27 levels and increased severity of shock associated with poor outcomes. Mice were then given norepinephrine or epinephrine, two catecholamines of the autonomic nervous system that are known to facilitate cross-talk between the immune and nervous system. The receptors (e.g., adrenoceptors) for norepinephrine or epinephrine are expressed on many different immune cells, including macrophages and DCs. Through inhibition of JNK signaling and IL-10 feedback loops, treatment with an adrenoceptor β_2_ agonist decreased levels of IL-27 and lessened the severity of shock in the mice [89].

### Human immunodeficiency virus

While IL-27 is also known to play a role in HIV type-1 infections, it has been suggested that there are no significant differences in IL-27 levels between HIV-infected and -uninfected populations [90]. Although endogenous IL-27 levels do not appear to differ between the two populations, recent studies suggest that modulation of these levels may be key in attenuating viral replication [91,92]. For example, a study by Garg *et al.* demonstrated a multifaceted role of IL-27 through the cytokine’s regulation of IFN-γ and IL-10 during a cytomegalovirus infection, commonly acquired by late-stage HIV patients. Specifically, IL-27 regulated increased expression of the T cell suppressive molecule B7-H4 on HIV^+^ MDSCs while also suppressing IL-10 production from CD4^+^ T cells [93]. In human monocyte-derived DCs, IL-27 exposure inhibited HIV replication. However, unlike its role in HIV^+^ macrophages and T cells, IL-27 inhibited HIV via an IFN-γ-independent mechanism [94]. This evidence suggests the potential therapeutic utility of IL-27 as an antiviral treatment for HIV patients.

## Conclusion & future perspective

In summary, IL-27 is a pleiotropic cytokine with established anti- and pro-inflammatory effects that are dependent upon cell-type and infectious context. It has direct influence on both the innate and adaptive arms of the immune response, while acting as a bridge between the two. On the innate side, IL-27 can manipulate various immune mechanisms such as phagolysosomal acidification, oxidative burst and subsequent ROS release and IFN-γ-induced autophagy. Within adaptive immunity, IL-27 modulates multiple T cell subsets, including Th1, Th2, Th17 and Foxp3^+^ Treg cells. Interference with IL-27 signaling by genetic deletion or neutralizing reagents results in increased bacterial killing. However, long-term interruption of IL-27 signaling can have important consequences to host survival. Moving forward, further research to understand the sometimes divergent IL-27 signaling during different infectious conditions is warranted to develop IL-27 immunomodulation as a translational application. Special attention should be given to the timing of IL-27 modulation, as maintaining the critical balance between Th1 and Th2 immunity is necessary to ensure protective inflammation and subsequent negative regulation at the appropriate time during an infection.

**Executive summary**

- IL-27 is a pleiotropic cytokine with both pro-inflammatory and anti-inflammatory activity that signals primarily through JAK-STAT and p38 MAPK/ERK pathways.
- IL-27 signaling modulates innate immune cells, such as professional antigen-presenting cells (monocytes, dendritic cells and macrophages) as well as microglia and neutrophils.
- IL-27 exerts its effects in a cell-dependent manner, wherein it more commonly exerts pro-inflammatory effects on monocytes and anti-inflammatory effects on macrophages and dendritic cells.
- Non-antigen-presenting cells cells, such as endothelial and epithelial cells, as well as fibroblasts can respond to IL-27 and act in concert with the innate immune response.
- IL-27 levels are increased by direct host cell interactions with bacteria, viruses and parasites and infectious outcomes are determined in a pathogen-specific manner.
- IL-27 negatively regulates phagocytosis, oxidative burst and autophagy in innate immune cells.
- IL-27 levels are higher in neonates relative to adults; macrophages and myeloid-derived suppressor cells are the dominant source of IL-27 in this population.
- Immunomodulation of IL-27 may offer promise for infectious diseases such as sepsis, pathogen insults to the central nervous system and peripheral nervous system, tuberculosis and HIV.
